# CORRELATES OF HEALTH LITERACY IN THE BLACK BELT AREAS OF ALABAMA: IMPORTANCE OF INTERNET ACCESS

**DOI:** 10.1093/geroni/igac059.1916

**Published:** 2022-12-20

**Authors:** Avani Shah, Yan Luo, LeAnna Roberts, Hee Lee

**Affiliations:** University of Alabama, Tuscaloosa, Alabama, United States; The University of Alabama, Tuscaloosa, Alabama, United States; University of Alabama, Tuscaloosa, Alabama, United States; University of Alabama, Birmingham, Alabama, United States

## Abstract

Health literacy, which is defined as being able to understand and utilize information related to one’s health, is an essential part of the health care process as it is related to health outcomes. However, little is known about health literacy in Black Belt communities in Alabama although this rural area has shown very high rates of health concerns such as diabetes, cardiovascular disease, stroke, and cancer (CDC Interactive Atlas, 2022). Study participants were recruited from the Black Belt areas of Alabama. A total of 180 African-American participants with a mean age of 57.5 completed a survey. A regression analysis was conducted to understand if sociodemographic and other relevant factors would predict health literacy. Lower education, gender, age, perceived racism, and perception that race impacts health care quality predicted health literacy. Interestingly, those with less internet access had significantly lower health literacy. Access to internet and smart phones was a reported concern for over a third of the participants. Only a quarter of participants reported use of the internet to assist with lifestyle modifications while almost half reported internet use to obtain health information; suggesting this as a potential means to improve health literacy and even potential lifestyle modification in health behaviors. Recommendations are made for interventions to improve health literacy in minority populations of this underserved region.

